# The Structure of the Talin/Integrin Complex at a Lipid Bilayer: An NMR and MD Simulation Study

**DOI:** 10.1016/j.str.2010.07.012

**Published:** 2010-10-13

**Authors:** Antreas C. Kalli, Kate L. Wegener, Benjamin T. Goult, Nicholas J. Anthis, Iain D. Campbell, Mark S.P. Sansom

**Affiliations:** 1 Department of Biochemistry, University of Oxford, South Parks Road, Oxford OX1 3QU, UK; 2 University of Leicester, Department Biochemistry, Room 1/40, Henry Wellcome Building, Leicester LE1 9HN, UK

## Abstract

Integrins are cell surface receptors crucial for cell migration and adhesion. They are activated by interactions of the talin head domain with the membrane surface and the integrin β cytoplasmic tail. Here, we use coarse-grained molecular dynamic simulations and nuclear magnetic resonance spectroscopy to elucidate the membrane-binding surfaces of the talin head (F2-F3) domain. In particular, we show that mutations in the four basic residues (K258E, K274E, R276E, and K280E) in the F2 binding surface reduce the affinity of the F2-F3 for the membrane and modify its orientation relative to the bilayer. Our results highlight the key role of anionic lipids in talin/membrane interactions. Simulation of the F2-F3 in complex with the *α/β* transmembrane dimer reveals information for its orientation relative to the membrane. Our studies suggest that the perturbed orientation of talin relative to the membrane in the F2 mutant would be expected to in turn perturb talin/integrin interactions.

## Graphical abstract

**Graphical Abstract:**

**Figure:**
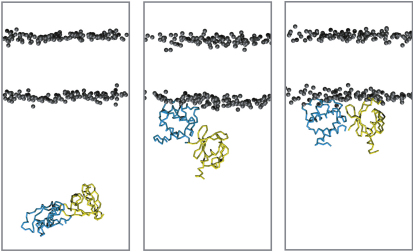

**Highlights:**

► Simulations and NMR define the interactions of the talin head domain with membranes ► Talin binds to anionic lipids in a bilayer via the F2 subdomain ► Mutations in the F2 lipid-binding site may perturb talin/integrin interactions

## Introduction

Integrins are heterodimeric cell surface receptors that link the exterior and interior of a cell. They are central components of focal adhesion complexes that form during cell migration, and are crucial for a variety of signal transduction events such as proliferation, survival/apoptosis, differentiation, and gene expression (Hynes, 2002). They are comprised of an α and β subunit, each containing an 80–150 kDa extracellular domain, a transmembrane (TM) domain, and a largely unstructured cytoplasmic domain (Wegener and Campbell, 2008). In mammals there are 18 α and 8 β subunits. These can heterodimerize to form 24 distinct integrins with specific functions. Integrins transmit signals in either direction across the cell membrane. Via the “outside-in” activation pathway the engagement of extracellular matrix ligands can cause changes to the cytoplasmic region resulting in downstream signaling events (Hynes, 2002; Schwartz and Ginsberg, 2002). In the “inside-out” activation pathway the binding of talin to the membrane proximal region of the β subunit is a key step of integrin activation (Bouaouina et al., 2008; Calderwood, 2004; Calderwood et al., 1999; Petrich et al., 2007; Tadokoro et al., 2003; Ulmer et al., 2003; Wegener et al., 2007).

Talin consists of a rod domain (Critchley and Gingras, 2008) and a head domain. The head domain can be broken down into four subdomains: F0, F1, F2, and F3. Integrin activation is mediated via the talin head domain, with the F3 domain being sufficient for activation of the integrin αIIbβ3 (Calderwood et al., 2002). One current activation model suggests that when the talin head domain binds to the integrin β cytoplasmic tail, separation of the two TM helices of the integrin occurs, leading to conformational changes in the ectodomain and a consequent increase in the affinity of the integrin (i.e., activation) (Kim et al., 2003; Lu et al., 2001; Luo et al., 2004, 2007; Partridge et al., 2005). During activation, it is suggested that a positively charged surface of the talin head domain is directed toward the cytoplasmic surface of the membrane, and that interactions between the talin and negatively charged (i.e., acidic) headgroups of membrane lipids occur (Anthis et al., 2009; Wegener and Campbell, 2008). These interactions are believed to play a crucial role in the inside-out activation process.

Recent experimental studies have revealed structures for talin integrin tail complexes (Anthis et al., 2009; Garcia-Alvarez et al., 2003) and for the TM region of the integrins (Lau et al., 2009; Vinogradova et al., 2002; Yang et al., 2009). Furthermore, several studies have highlighted the importance of the interactions between the cytoplasmic region of the β tail of the integrin and talin head domain in integrin activation (Hughes et al., 1995; Ulmer et al., 2003; Vinogradova et al., 2002; Wegener et al., 2007). However, the importance of the interactions between talin and the membrane in integrin activation is relatively poorly understood and a more detailed characterization is required.

**Figure 1:**
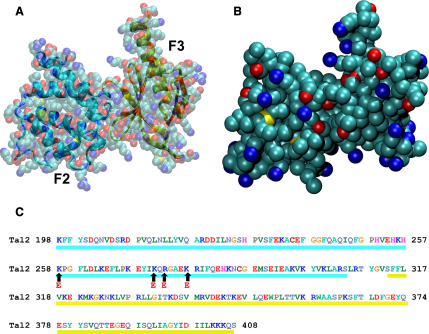
The Talin F2-F3 Domain (A) Atomistic structure (shown in cartoon and VDW formats) and (B) coarse-grained (CG) model of the F2-F3 domain. In the atomistic model the F3 domain is shown in yellow and the F2 in cyan. In the VDW model (transparent) the colors for atoms are: cyan = carbon; red = oxygen; blue = nitrogen; yellow = sulfur. In the coarse-grained model the colors for CG particles are: cyan = apolar; red = polar; blue = positively charged; bronze = negatively charged; yellow = neutral. (C) Sequence of the talin2 F2-F3 domain with the mutated residues indicated by arrows. The F2 domain is underlined in cyan and the F3 domain in yellow.

Here, we investigate the interactions between a lipid bilayer and talin, and attempt to identify their role in integrin activation by combining experimental and computational techniques. In particular, nuclear magnetic resonance (NMR) and coarse-grained molecular dynamics (CG-MD) simulations are used to explore the position, orientation, and interactions of wild-type and mutated (at residues K258E, K274E, R276E, and K280E) talin2 F2-F3 and F2 domains (Figure 1), with different neutral (i.e., zwitterionic) and anionic lipids. To the best of our knowledge, this is the first simulation of the complex of the talin2 F2-F3 domains and the integrin α and β TM helices. The results provide novel information concerning the orientation of talin relative to the bilayer and to the two helices. A good correlation between experimental and simulation results was obtained, and it is shown that mutations of positively charged residues in the F2 domain affect the orientation of talin with respect to the membrane providing new insights into F2-F3/membrane interactions and their role in the integrin activation mechanism.

## Results

### NMR Studies of Talin/Bilayer Interactions

#### F2-F3/Bilayer Interactions

**Figure 2:**
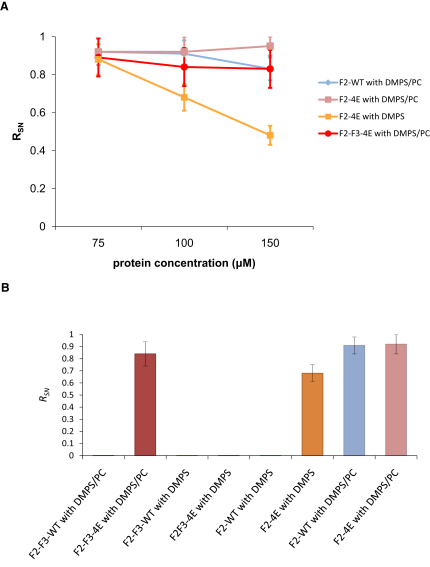
Protein/Liposome Interactions by NMR (A) Protein/liposome titrations. The ratio *R_SN_* (see Experimental Procedures for details) is shown for F2-F3-4E with 20% DMPS/PC liposomes (purple), for F2-4E with DMPS liposomes (orange), for F2-WT with DMPS/PC liposomes (blue), and for F2-4E with DMPS/PC liposomes (red). Values close to 1 imply weak interaction with the liposomes, whereas values near zero imply strong interactions. (B) The value of *R_SN_* is compared for the different proteins at a concentration of 100 μM. The same color code as in the previous figure is used. The ratio *R_SN_* is mean value ± standard deviation from the signal/noise ratio with and without the liposomes for every peak in each system. See also Figure S1 and Figure S2.

It was recently suggested that a positively-charged patch on the surface of the talin F2 domain interacts with the membrane, and furthermore that mutations in residues K258, K274, R276, and K280 in talin2, or K256, K272, K274, and R277, in talin1 disrupt these interactions (Anthis et al., 2009). To test this hypothesis, the F2-F3-WT (or F2-WT) and the F2-F3-4E (or F2-4E) (i.e., K258E, K274E, R276E, and K280E) domains were added to liposomes and their chemical shift perturbations were monitored in ^1^H-^15^N-HSQC NMR spectra. Three different protein concentrations were used (75 μM, 100 μM, 150 μM; see Figure 2A), indicating the reproducibility of our results. For each concentration two spectra were obtained; a “test” spectrum with the protein alone and a spectrum of the protein in the presence of negatively charged liposomes. The induced shifts were very small at all three protein concentrations. Intensity changes were therefore used to detect binding rather than shifts. The two spectra were used to calculate a parameter to calculate a parameter *R_SN_*, which was calculated as:

$$R_{SN}=\left(S_{protein\;with\;liposome}\right)/\left(S_{protein\;without\;liposome}\right),$$

where *S* is the signal/noise ratio for a given spectrum. As a protein binds more tightly to a liposome the correlation time increases due to the vastly increased molecular weight of the complex, the spectral peaks broaden and *R_SN_* becomes smaller (see Experimental Procedures for further details). *R_SN_* was thus used as an indication of interaction strength.

For F2-F3-WT in the presence of 20% DMPS/PC (1,2-dimyristoyl-*sn*-glycero-3-phospho-L-serine/L-α-phosphatidylcholine) liposomes (i.e., mixed anionic/neutral), no ^1^H-^15^N-HSQC spectrum was observed, i.e., *R_SN_* ≈ 0 implying that F2-F3-WT bound tightly to the liposomes. However, it was possible to get an ^1^H-^15^N-HSQC spectrum (*R_SN_* ≈ 0.8) (Figure 2B) from F2-F3-4E indicating that loss of the four basic residues from the surface of the F2 domain reduced binding to the liposomes.

Similar experiments were carried out for F2-F3 with pure DMPS (1,2-dimyristoyl-*sn*-glycero-3-phospho-L-serine) liposomes. In this case *R_SN_* ≈ 0 for both the WT and the 4E mutant. This indicates that the strength of interaction of F2-F3 is increased as the fraction of anionic lipid is increased. In vivo the fraction of anionic lipid in the inner leaflet of mammalian plasma membranes is ∼20 mol % (Yeung et al., 2006), similar to our 20% DMPS/PC condition.

#### F2/Bilayer Interactions

We also explored the interaction of the WT and F2-4E domains with liposomes. For the F2-WT domain in the presence of DMPS liposomes no spectrum was observed at any protein concentration (i.e., *R_SN_* ≈ 0), implying a strong F2-WT interaction with the liposomes. In contrast, in the ^1^H-^15^N-HSQC spectra of the F2-4E with and without DMPS liposomes, the shift induced in the peaks was very small (see Figure S1 and S2 available online), suggesting that the F2-4E mutations in the F2 domain inhibited binding to the negatively charged lipids. Interestingly the *R_SN_* value for F2-4E with DMPS liposomes (0.68) is lower than the *R_SN_* for F2-4E (0.92) and for F2-F3-4E with 20% DMPS/PC (0.84). Given the qualitative nature of the results, this suggests that the interaction of the F2 domain with DMPS liposomes can be classified as “strong.” In contrast, *R_SN_* for the interaction of the F2 with 20% DMPS/PC liposomes is larger for both F2-WT and F2-4E than for the other systems. Therefore the interaction of F2 with 20% DMPS/PC can be classified as “weak.” By similar criteria, the interaction of the F2-F3 with 20% DMPS/PC liposome can be classified as “intermediate” and with DMPS as “very strong.” It is important to note that there is therefore some residual interaction of the protein with the bilayer for the F2-F3-4E mutant. This may reflect either nonproductive binding of F2 (see below) and/or interaction of F3 with the bilayer.

### MD Simulations

Having shown that the binding of the talin F2-F3 domain to bilayers depends on the interaction between anionic lipids and basic side chains in F2, we used CG-MD simulations (summarized in Table 1) to develop a molecular model of these interactions, and to explore the possible role of interactions with the β-integrin TM domain.

#### F2-F3/Bilayer and F2/Bilayer Interactions

**Figure 3:**
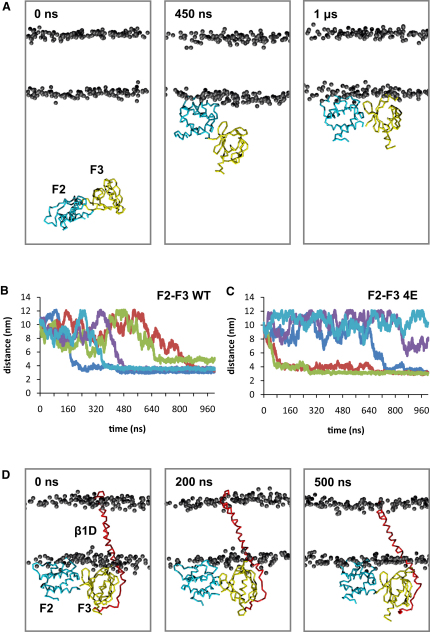
F2-F3 Simulations (A) The F2-F3 simulation. Snapshots of the simulation systems are shown at 0 ns, 450 ns and 1 μs. The F2 domain is shown in cyan, and the F3 domain in yellow. The bilayer lipid phosphates are in gray. The lipid tails and waters are omitted for clarity. (B and C) Progress of the F2-F3 and F2-F3-4E simulations, respectively (five of each, indicated by the differently colored lines) are shown as the distance of the center of mass of the protein from the center of mass of the bilayer as a function of time. (D) Snapshots from the F2-F3-β1D simulations with the β1D subunit in red and other colors as in (A).

In the first set of simulations we compared the binding of WT and F2-F3-4E with a preformed POPC/POPG (palmitoyl-2-oleoyl-*sn*-glycero-3-phosphocholine/palmitoyl-oleoyl-phosphatidyl glycerol at a ratio of 3:2; i.e., mixed zwitterionic/anionic) lipid bilayer. Five simulations were run with the F2-F3 domain initially displaced 100 Å away from a bilayer (Figure 3A) for both the WT and the mutant. In all five simulations the WT domain initially diffused in the aqueous environment before binding to the bilayer at the lipid/water interface; it then remained bound for the remainder of the 1 μs simulation (Figures 3A and 3B). In contrast, for the 4E mutant only three simulations out of five resulted in binding of F2-F3 to the bilayer (Figure 3C) consistent with a weakened talin/membrane interaction.

**Figure 4:**
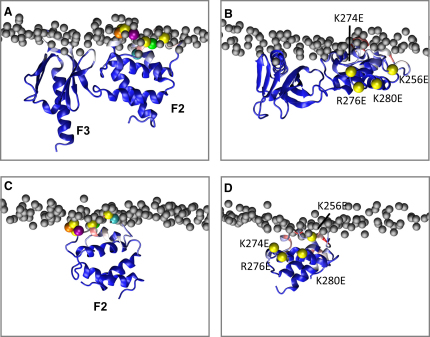
Lipid Contacts of the Protein Contacts between the F2-F3 domain (A and B) and the F2 domain (C and D) and lipids. In (A), the contacts seen in the preferred orientation of the F2-F3 domain are shown as the backbone particles of the residues that made the highest number of contacts with the lipids: L262 (green), E266 (cyan), Q275 (orange), and G277 (purple). The yellow spheres correspond to the key basic residues (K258, K274, R276, and K280) in F2. In (B) the F2-F3-4E simulation is shown with the mutated residues (258, 274, 276, and 280) again shown in yellow. The phosphate groups are shown as gray spheres. Corresponding diagrams for the F2 and F2-4E simulations are shown in (C) and (D) respectively. For F2 the residues showing the highest number of contacts are: K256 (cyan), L262 (green), Q275 (orange), and G277 (purple). In each case a distance cutoff of 8 Å between the outermost side-chain particle and any lipid particles was used to define a contact.

For the F2-F3-WT simulations, the pattern of interacting residues in the preferred binding surface (this appeared in three of five simulations) is consistent with the published F2-F3-β1D complex structure (Anthis et al., 2009; see below). Thus, lysine and arginine residues of the F2 subdomain of F2-F3 make large numbers of contacts with the lipids (Figure 4A). In contrast, in the three F2-F3-4E mutant simulations that yielded a complex, a different pattern of interactions between the protein and the bilayer was observed (Figure 4B). Therefore, the 4E mutations of F2 not only reduce the affinity of F2-F3 for the membrane but they also perturb the orientation of protein relative to the membrane when bound. This, in turn, might be anticipated to perturb talin/integrin interactions.

We also compared the interaction of the isolated F2 WT domain and 4E mutant with a preformed POPC/POPG bilayer. The F2 WT bound to the bilayer in all five simulations carried out. The preferred binding surface (appearing in three of five simulations) (Figure 4C) was consistent with the F2-F3-WT simulations (above) and the structure of the F2-F3-β1D complex (Anthis et al., 2009). In contrast, simulations of the F2-4E mutant with a lipid bilayer did not yield the preferred binding surface in any of the four of five simulations in which the F2-4E finally bound the bilayer (Figure 4D). As for F2-F3, this suggests that the mutations inhibit the F2 domain from interacting productively with the bilayer.

We have also carried out control simulations (for both F2-F3 and for F2) in which we made the quadruple K258E, K274E, R276E, and K280E mutation but set the charge on the four glutamate side chains to zero (this may be viewed as an approximation to setting these four side chains to a protonated state). For both F2 and for F2-F3 this yielded only nonproductive complexes (i.e., the protein bound to the bilayer), but with a different orientation from the preferred orientation in the wild-type. Thus the K/R motif in F2 is needed for the productive binding mode of the protein to be adopted.

Together, these four sets of simulations suggest a strong electrostatic interaction between the basic surface of the F2 subdomain and anionic lipid headgroups. The pattern of interacting lysine and arginine residues is conserved between the F2 and F2-F3 simulations and residues L262, D263, E266, Q275, G277, and R281 form the preferred surface made the largest number of contacts in both simulations.

#### The Talin F2-F3/Integrin β1D TM Complex in a Bilayer

We wished to extend our analysis of talin/bilayer interactions to include the possible reciprocal effects of the interaction of talin F2-F3 with the TM domain and intracellular juxtamembrane region of integrin β. This was possible because of the known X-ray structure (albeit in the absence of a lipid bilayer) of F2-F3 bound to the β1D TM helix and tail (Protein Data Bank [PDB] 3G9W). We therefore took the F2-F3-β1D structure (converted to a CG structure) and positioned it in a preformed POPC/POPG bilayer with the β1D TM helix oriented such that the positively charged patch of F2-F3 interacted with the membrane surface; this resulted in the TM helix spanning the bilayer with a tilt angle of ∼15°. This F2-F3-β1D/bilayer complex was then simulated for 0.5 μs (Figure 3D). During the simulation, the β1D tilt angle increased by ∼20° to a final value of ∼35°. This increase in β1D tilt angle might be crucial in disrupting the interaction between the two integrin tails and lead to tail separation and integrin activation.

**Figure 5:**
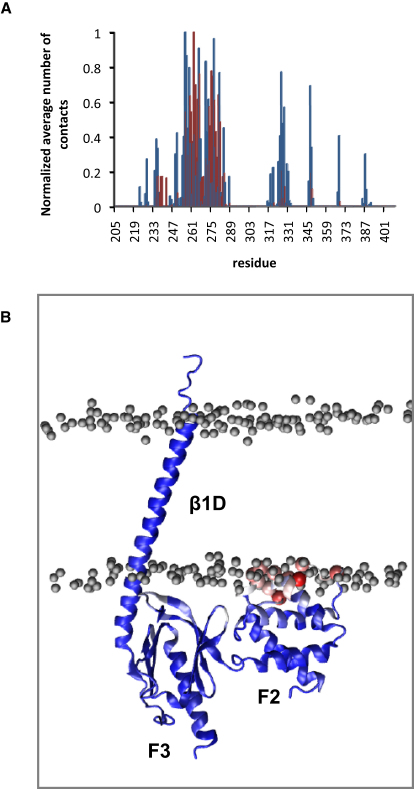
The F2-F3-β1D Simulation (A) Normalized average number of contacts as a function of residue number for the F2-F3 domain and lipids in the F2-F3-β1D simulation. Red lines correspond to the contacts between the lipid headgroups and the F2-F3 domain, and blue lines to the contacts between the lipid tails and the protein. (B) Snapshot, corresponding to the last frame of the simulation, of the F2-F3-β1D simulation system. The normalized average number of lipid/protein contacts was mapped onto the structure and is shown on a color scale from blue (low number of contacts) through white to red (high number of contacts).

The F2-F3/lipid interactions in the pre-inserted F2-F3-β1D simulations were compared (Figure 5). For this analysis, the number of contacts (as defined by a distance cut-off of 8 Å [Hall and Sansom, 2009]) between the protein residues and the lipids were counted over the last 50 ns of each simulation, and then normalized. Residues L262, D263, E266, Q275, G277, and R281 appeared to form a significant number of contacts with the lipids, in addition to the strong interactions between the lipids and the surface lysines and arginine residues in the talin F2 domain. Interestingly, the same residues (i.e., binding surface) formed a significant number of contacts with the bilayer in the simulations with F2-F3-WT and F2-WT (above). Significantly, if one analyses the lipids in contact with protein e.g., the F2-F3 simulations, the ratio of PC:PG contacts is 1:2, despite an overall bilayer composition of 3:2. Thus the protein binding region of the bilayer is enriched in anionic lipids about 3-fold relative to the bilayer as a whole.

#### Self-Assembly Simulations with the Talin F2-F3/Integrin αIIb-β1D TM Complex

**Figure 6:**
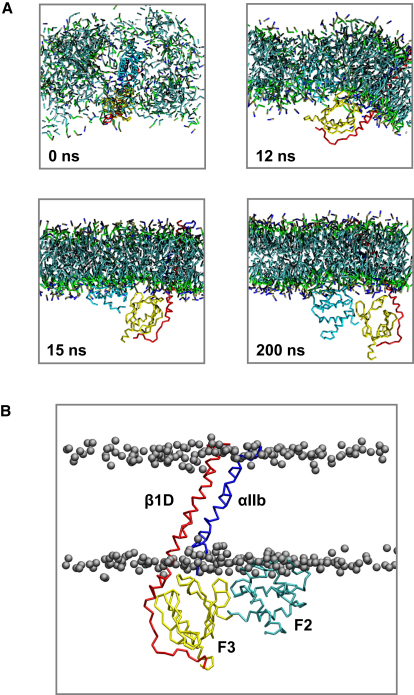
The F2-F3-αIIb-β1D Simulation (A) Progress of the bilayer self-assembly simulation for F2-F3-αIIb-β1D. In the initial setup, randomly placed lipids (POPC and POPG at a ratio of 3:2, shown in bonds formats with the hydrophobic tails in cyan, glycerol in green, phosphate in brown, and choline in blue) surround the protein complex (F2 in cyan, F3 in yellow, αIIb in blue, β1D in red). Waters and counter ions are omitted for clarity. (B) Final frame (200 ns) of a F2-F3-αIIb-β1D- SA simulation.

We extended our study to a self-assembly simulation (Figure 6) of lipids with a model of the complex formed by the F2-F3-WT domain bound to the αIIb-β1D TM integrin helix heterodimer. This model was generated by adding the β1D TM domain and the αIIb TM and cytoplasmic domains to the F2-F3-β1D X-ray structure (PDB 3G9W) (Anthis et al., 2009), based on the NMR structure (PDB 2K9J) (Lau et al., 2009) of the α/β TM heterodimer. The CG-MD self-assembly protocol allows us to explore the orientation of a complex membrane protein in a lipid bilayer without assuming an initial location for the protein (Scott et al., 2008). Note that the elastic network model used in this simulation does not permit dissociation of the α and β subunits. In the self assembly simulation, we observed that the protein complex reached a final position and orientation in the lipid bilayer identical to that observed in simulations of the interaction of F2, F2-F3, and F2-F3-β1D discussed above. The β1D tilt angle was ∼27°.

To test the effect of the 4E mutations on the F2 binding surface, the self assembly simulations were repeated for F2-F3-4E-αIIb-β1D. Ten separate CG-MD self-assembly simulations were carried out. However, in none of them did F2-F3-4E reach a final binding surface similar to the “correct” binding surface observed in the WT simulations of F2, F2-F3, and of F2-F3-αIIb-β1D. Thus the self-assembly simulations on the complete complex support the picture obtained from NMR and simulation studies of the talin subdomains that electrostatic interactions between the surface of F2 and anionic lipids drive the talin/integrin interaction with a cell membrane.

## Discussion

A combination of experiment and simulation has revealed the nature of the interactions of the talin F2-F3 domain with a lipid bilayer membrane. We have defined residues within the F2 domain that play a key role in interactions with negatively charged lipid headgroups of the bilayer. Thus, four mutations (K258E, K274E, R276E, and K280E) in the binding surface of F2, which switch the net charge from +4 to −4, alter the nature of the interactions between talin and the bilayer. In the presence of these mutations the F2-F3 domain binds the membrane with a modified orientation that may be anticipated to perturb the interaction of the two TM helices of the integrin heterodimer.

Using NMR to characterize the interactions between talin and variously charged liposomes, it has been demonstrated that negatively charged headgroups in the bilayer promote the binding of talin to the membrane. Analysis of the simulations confirms this result because the number of contacts between either the F2-F3 domain or the F2 domain and the anionic headgroups of POPG is consistently larger than the number of contacts with the zwitterionic headgroups of POPC. In particular, the interactions of the F2-F3 domains were 3-fold higher with POPG/POPC than with POPC alone. In addition, the presence of F3 in combination with F2 seems to enhance the binding of talin to the membrane. Thus, in the presence of the F3 domain the protein is capable of binding to 20% anionic liposomes in the NMR studies, unlike the F2 subdomain alone. Therefore, anionic lipids in the bilayer and the presence of the F3 domain alongside the F2 domain are two key factors that enhance the interaction of talin with a membrane.

This is of likely mechanistic significance. It has been suggested (Anthis et al., 2009) that the formation of a salt bridge between the β integrin TM helix/tail with talin may be a crucial step of integrin activation. In particular, a salt bridge is thought to be formed between K327 of F3 in talin2 (or K324 in talin1) and D759 of β1D (or D723 in β3). This interaction requires formation of a stable talin F2-F3 complex with the bilayer, correctly oriented to interact with the β-integrin TM domain/tail. Our simulations of the binding of the mutant talin F2-F3-4E reveal a different orientation relative to the bilayer that might thus be expected to inhibit the formation of this talin/β tail salt bridge, and thus to inhibit integrin activation. It is known that disruption of the αIIb R995-β3 D723 salt bridge of the integrin heterodimer is not sufficient alone for integrin activation (Tadokoro et al., 2003; Wegener et al., 2007); rather talin binding to the β integrin tail is required to fully activate the integrin by replacing the αIIb R995-β3 D723 salt bridge with the talin/β1D bridge (Anthis et al., 2009). However, we should note that in our F2-F3-β1D coarse-grained simulations K327 of F3 and D759 of β1D were held in contact throughout the whole simulation by the elastic network model, and thus a multiscale approach (see below) may be needed to test the predicted destabilization of the interaction by the perturbed bilayer/talin interaction.

The surface of F2-F3 that binds to the membrane consists of a stretch of basic and acidic residues. Residues K258, K274, R276, and K280 appear to make a substantial number of contacts with anionic lipids in the simulations explaining why mutations in these residues disrupt interaction with the membrane. This binding surface is consistent with NMR (Wegener et al., 2007) and recent total internal reflection fluorescence microscopy studies (Saltel et al., 2009). It is significant that the correct orientation of F2-F3 at the bilayer was seen in our simulation without the β1D TM helix. This supports our suggestion that the F2/bilayer interaction positions F3 so as to facilitate interactions with β1D. A perturbed orientation of F2-F3 relative to the membrane (due to disruption of the talin/membrane interaction) would thus be expected to result in a less productive interaction of the F3 with β1D.

This study raises an interesting issue of relevance to ongoing efforts in integrative structural biology. It might be contended that visualization alone could reveal the likely bilayer interaction surface of F2-F3. However, such an approach simply enables one to formulate a hypothesis that may be subsequently testing using simulation. Indeed, a more rigorous computational approach to evaluation of structural hypotheses is essential if one is to use the outcomes of integrative structural biology studies (Alber et al., 2008) as platforms for further investigations into higher levels of biological organization and function. Thus, we have used simulations to test an initial structural hypothesis alongside experiments designed to validate the simulations.

From a more methodological perspective, this study demonstrates that CG-MD simulations may be used to predict the position and the orientation of a protein domain bound to a lipid bilayer. This builds on previous CG-MD studies of integral membrane proteins (Scott et al., 2008) and of membrane bound enzymes (Balali-Mood et al., 2008; Wee et al., 2008). In particular, the self assembly CG-MD simulations for F2-F3-αIIb-β1D reported here extend this general approach to more complex membrane/protein assemblies containing both transmembrane elements and domains that interact with the bilayer surface.

Of course, there are limitations to the accuracy of CG-MD simulations (Allen, 2007). To improve on this, we will extend the current studies to embrace multiscale simulations (Ayton and Voth, 2009), by converting the CG models back to atomistic resolution, allowing their refinement by conventional MD simulations. As has been seen for the S4 helix of Kv channels (Wee et al., 2010) for example, this allows for a more quantitative approach to protein/bilayer interactions.

## Experimental Procedures

### Protein Expression and Purification for NMR

U-^15^N-labeled F2, F2-4E, and F2-F3 domains were expressed as GST-fusion proteins in M9 minimal media using an ^15^N source. Cells were harvested by centrifugation and resuspended in phosphate-buffered saline. Lysozyme (1 mg/mL), 1 M MgSO_4_ (10 mL/mL), and 1 mg/mL DNase1 solution (10 μL/mL) were added. After three freeze/thaw cycles, 10% Triton was added and the sample was centrifuged at 40,000 × g for 10 min at 4°C. A glutathione-Sepharose 4B (Roche Applied Science) column was set up for purification and the protein was eluted in fractions of 15 mM glutathione in 50 mM Tris (∼pH 5.8). Glutathione S-transferase fusion protease 3C^pro^ was added to cleave the purified fusion protein. The protein was further purified by gel filtration chromatography into NMR buffer (50 mM sodium phosphate, 100 mM NaCl, 1 mM dithiothreitol [DTT], pH 7.0). The F2-F3-4E domain was expressed as a His-tag fusion protein and purified as mentioned above, but with Talon resin (Takara Bio) in 50 mM sodium phosphate, 300 mM NaCl, 0.035% β-mercaptoethanol, pH 7.0 buffer. TEV protease was used to cleave the polyhistidine tags and gel filtration chromatography into pH 7.0 NMR buffer was used for further purification.

### Liposomes

To construct the DMPS/PC 1:4 mol %, where DMPS is L-α-phosphatidylcholine/1,2-dimyristoyl-*sn*-glycero-3-phospho-L-serinenad and PC is L-α-phosphatidylcholine (egg, chicken) with predominant species 16:0/18:1, DMPS and PC lipids (in chloroform) were mixed at the required ratio. The chloroform was removed using a stream of argon gas and the sample was attached to a vacuum pump overnight. One milliliter of 50 mM phosphate, 100 mM NaCl, and 1 mM DTT was added to the liposomes and the sample was agitated. At 40°C the sample was rotated for 1 hr. The large multilamellar vesicles (LMVs) were disrupted by five freeze-thaw cycles and an Avanti mini-extruder kit was utilized to extrude the sample to size the liposomes, following the protocol specified on the Avanti web site (http://avantilipids.com/) that states that unilamellar liposomal suspensions with a low polydispersity can only be prepared with membranes having a pore size of ≤200 nm. Accordingly, the suspension was extruded through a 100-nm polycarbonate membrane. The sample was maintained at a temperature of 40°C (i.e., above the phase transition temperature of the lipids). The sample was extruded back and forth 20 times. Vesicle suspensions were stored for a maximum of 3–4 days at 4°C to avoid any significant change in vesicle size distribution. The DMPS liposomes were constructed by using the same procedure as described above.

### NMR Spectroscopy

NMR experiments were carried out at 40°C on 600 MHz NMR spectrometers with Oxford Instruments superconducting magnets and GE Omega consoles. All experiments with both wild and mutated F2-F3 and F2 constructs with and without liposomes were carried out using samples of 75, 100, and 150 μM of U-^15^N-labeled protein in 5% D_2_O/95% H_2_O. All samples were buffered with 50 mM phosphate and 100 mM NaCl. In addition, 5 mM DTT was added to prevent oxidation. The samples with liposomes contained 10 mM lipids. All samples were set to pH 6.5. For each sample a two-dimensional heteronuclear single quantum coherence (HSQC) spectrum was acquired. The resonance assignments of the F2 domain were made using three-dimensional (3D) gradient-enhanced f-NOESY-HSQC (τ_m_ = 100 ms) and {^1^H-^15^N}-TOCSY-HSQC (τ_m_ = 70 ms) spectra. The NMR assignments of the F2-F3 domains and F2 domain that were used in this study have been submitted to the Biomagres Bank: F2 (196-309), BMRB ID = 16930 F2-F3 (196-405), BMRB ID = 169n32. NMR experiments were processed using NMRpipe (Delaglio et al., 1995) and visualized using SPARKY (www.cgl.ucsf.edu/home/sparky). The ratio *R_SN_* that characterizes the strength of the interactions, was calculated using the equation

$$R_{SN}=\frac{S_{protein\;with\;liposome}}{S_{protein\;without\;liposome}},$$

where *S* is the signal to noise ration for each spectrum. The signal to noise was calculated using NMRPipe and Sparky (www.cgl.ucsf.edu/home/sparky). Individual peak *R_SN_* values were calculated using the signal/noise ratio of the peak concerned, with and without liposomes. The overall *R_SN_* value for a particular condition, was calculated as the mean of the individual peak values ± the standard deviation. For the *R_SN_* calculations, 106 and 201 peaks for F2 and F2-F3, were used respectively.

### CG-MD Simulations

The crystal structure of F2-F3 bound to the β1D TM and tail domain (PDB 3G9W) and the structure of the αIIbβ3 complex (PDB 2K9J) were converted to a CG representation (Bond et al., 2007, 2008; Bond and Sansom, 2006) providing initial structures for the simulations. The CG simulations used a local variant (Bond et al., 2008) of the MARTINI (Monticelli et al., 2008) coarse-grained force field in which there is an approximate 4:1 mapping of heavy atoms to CG particles. The secondary and tertiary structure of the protein domains was modeled as an elastic network by imposing a harmonic force constraint (force constant = 10 kJ/mol/Å^2^) (Atilgan et al., 2001) between all backbone particles that were within a cutoff distance of 7 Å.

In the CG-MD simulations with the preformed POPC/POPG bilayer, the F2-F3 or F2 domain was displaced 100 or 80 Å away from a preformed bilayer containing POPC and POPG at a ratio of 3:2 (154 POPC and 102 POPG molecules). Each system was solvated with CG water particles and with CG sodium particles to neutralize the system. The system was energy minimized, and after 5 ns equilibration (with restraints applied to Ca atoms, force constant = 10 kJ/mol/Å^2^); production simulations were run for 0.5 ns and 1 μs respectively for F2 and F2-F3. Five simulations were carried out for each system with all simulations starting from the same configuration but with different initial velocities. The wild-type and the mutated protein simulations of each system started from the same initial configuration. In the self-assembly simulations, POPC and POPG lipids at a ratio of 3:2 (154 POPC and 102 POPG) were randomly positioned in a cubic box along with the protein, CG water particles, and counterions. The simulation was run for 200 ns after an initial 400 steps of energy minimization.

All CG-MD simulations were carried out using GROMACS 3.3.3 (www.gromacs.org) (Lindahl et al., 2001; van der Spoel et al., 2005). A Berendsen thermostat (Berendsen et al., 1984) was used for temperature coupling with a coupling constant of 1.0 ps. The reference temperature was 310 K. Electrostatic/Coulombic interactions utilized a relative dielectric constant of 20. Lenard-Jones and electrostatic/Coulombic interactions were shifted to zero between 9 Å and 12 Å and 0 Å and 12 Å, respectively. A Berendsen barostat with a coupling constant of 1.0 ps, a compressibility value of 5.0 × 10^−6^ bar^−1^ and a reference pressure of 1 bar was used. The integration time step was 40 fs and coordinates were saved and analyzed every 400 ps. Visualization was via VMD (Humphrey et al., 1996).

## Figures and Tables

**Table 1:** Summary of CG Simulations

Simulation	System	Particles (n)	Duration (ns)	Final RMSD^c^ (Å)
F2	F2-WT + 10289 W + POPC/POPG bilayer^a^	13,955	5 × 500	0.20 ± 0.02
F2-4E	F2-4E + 10243 W + POPC/POPG bilayer^a^	13,921	5 × 500	0.18 ± 0.03
F2-F3	F2-F3-WT + 13145 W + POPC/POPG bilayer^a^	17,020	5 × 1000	0.28 ± 0.05
F2-F3-4E	F2-F3-4E + 13095 W + POPC/POPG bilayer^a^	16,982	5 × 1000	0.29 ± 0.07
F2-F3-β1D	F2-F3-WT-β1D + 12047 W + POPC/POPG bilayer^a^	16,066	500	0.28
F2-F3-αIIb-β1D-SA	F2-F3-WT-αIIb-β1D + 5858W + POPC + POPG b	10,785	200	0.14
F2-F3-4E-αIIb-β1D-SA	F2-F3-4E-αIIb-β1D + 5774W + POPC + POPG b	10,734	10 × 200	0.13 ± 0.01

CG: course-grained; RMSD: root-mean-square deviation.

a These simulations used a preformed lipid bilayer.

b These simulations used self-assembly to form the bilayer in the presence of the protein, starting with randomly positioned and oriented lipid molecules.

c The RMSD was calculated over the whole trajectory for each simulation using the backbone particles, and measured relative to the starting structure for that simulation. After calculating the RMSD for all repeats of simulation of a given system, the mean value of the RMSD was calculated.

## Supplemental Information

Document S1. Two Figures
