# Supplementary material for: The Structure of the Talin/Integrin Complex at a Lipid Bilayer: An NMR and MD Simulation Study
Source: Structure. 2010 Oct 13;18(10):1280–8. doi: 10.1016/j.str.2010.07.012 (PMC3032884; doi:10.1016/j.str.2010.07.012)
Supplement: Document S1. Two Figures [file mmc1.pdf]

Structure *18*

Supplemental Information

The Structure of the Talin/Integrin Complex at a Lipid Bilayer: An NMR and MD Simulation Study

Antreas C. Kalli, Kate L. Wegener, Benjamin T. Goult, Nicholas J. Anthis, Iain D. Campbell, and Mark S.P. Sansom

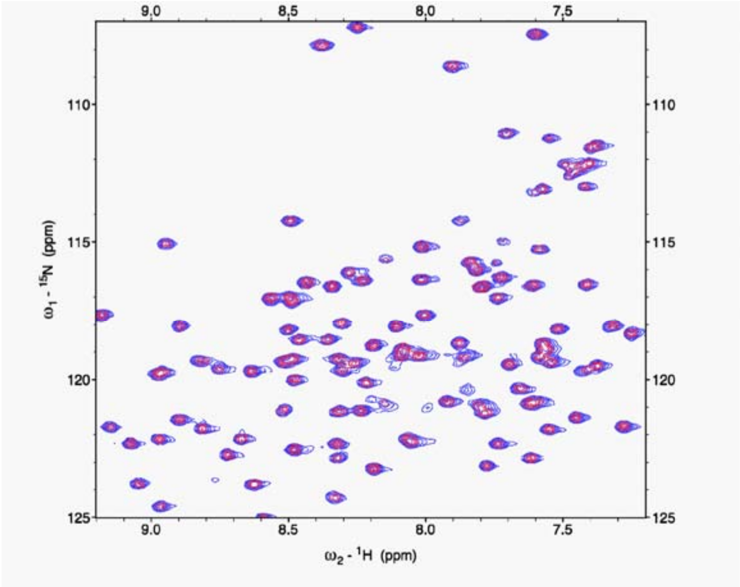

Figure S1, related to Figure 2:
Overlaid ^{1}H-^{15}N-HSQC NMR spectra of the F2-4E domain in the absence (blue) and F2-4E in the presence (red) of DMPS liposomes at a concentration of 100 µM. The figure shows that the shifts of the peaks induced by the addition of DMPS liposomes were very small.

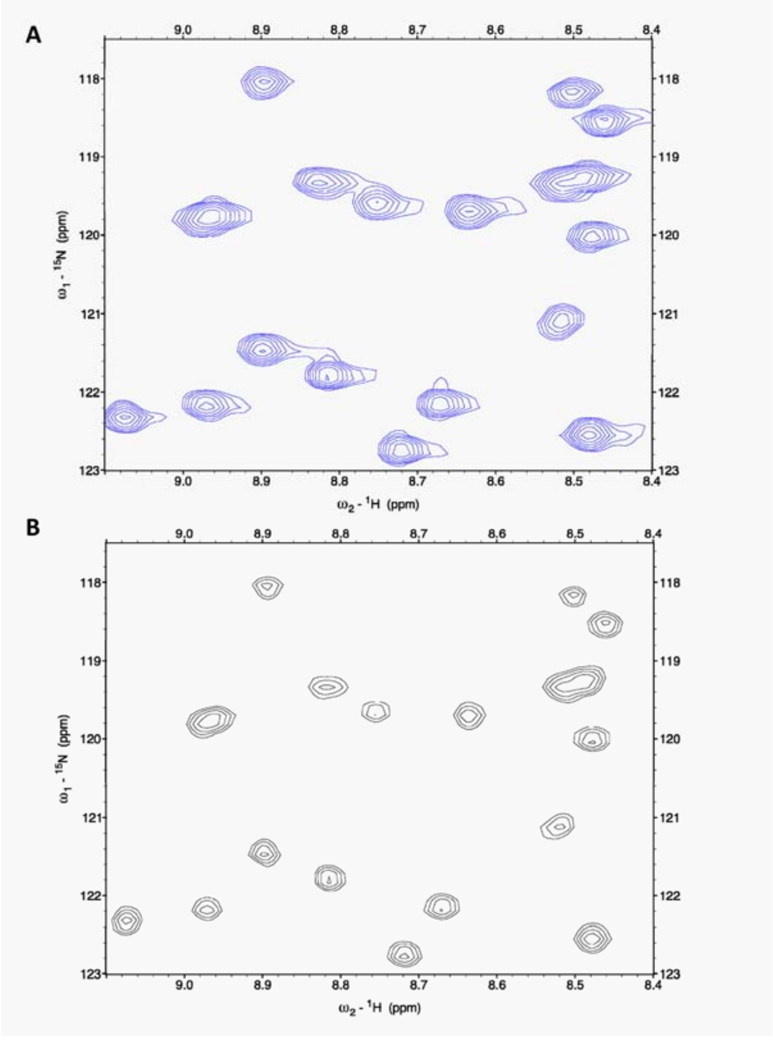

Figure S2, related to Figure 2:
^{1}H-^{15}N-HSQC NMR spectra of the F2-4E **A** in the absence of and **B** in the presence of DMPS liposomes (100 µM) showing the reduction of the intensities upon the addition of liposomes.
